# Obtaining and Characterization of New Materials

**DOI:** 10.3390/ma14216606

**Published:** 2021-11-03

**Authors:** Andrei Victor Sandu

**Affiliations:** 1 Faculty of Material Science and Engineering, Gheorghe Asachi Technical University of Iasi, 41 D. Mangeron St., 700050 Iasi, Romania; sav@tuiasi.ro; 2 Romanian Inventors Forum, 3 Sf. Petru Movila St., L11, III/3, 700089 Iasi, Romania; 3 National Institute for Research and Development for Environmental Protection INCDPM, 294 Splaiul Independentei, 060031 Bucharest, Romania

The main objective of this Special Issue was to publish outstanding papers presenting cutting-edge research in the field of new materials and their understanding.

At present, more and more obtaining procedures and technologies are available next to advanced characterization techniques. The Special Issue managed to gather several outstanding articles in a broad field, from obtaining new materials to their understanding using the latest characterization techniques.

In this way, the focus was on new materials used in the water treatments: one study allowed the selection, on the basis of the caustic module, of ceramics with a high capacity for ionic exchange [1]; and another focused on new ecological materials for replacing cement (geopolymers and cementitious materials), highlighting the thermal stability of geopolymers in the 25–1000 °C temperature range through the use of thermogravimetric analysis, differential thermal analysis, and XRD [2]. Other studies on geopolymer composite showed that temperatures exceeding 400 °C accelerated the strength development, thus increasing the strength of the DFA composites [3], respectively. The use of embedded biofilm-resistant photoactivated TiO_2_ nanoparticles at low concentrations in the cementitious composite matrix is an effective method to increase material durability and reduce maintenance costs [4]. All these published articles focused on materials which reduce the CO_2_ footprint [2,3,4].

A very interesting field of research is that of insulating materials, concluding that the sheep wool has a comparable sound absorption performance to mineral wool or recycled polyurethane foam [5]. For the first time, three new parameters of integration efficiency of the thermal insert, thermal insulation efficiency parameters, and efficiency parameters of the integration of the textile material integrated into the clothing system were introduced; based on these parameters, it was possible to perform an effective and accurate comparative analysis of the thermal insulation of multi-layer thermal inserts in clothing [6].

An interesting approach looked at biomaterials and medical applications, concluding that structural material flaws account for a high percentage of the observed causes of failure, and identifying the breaking mechanisms macroscopic and microscopic investigations, including stereomicroscopy and scanning electron microscopy, is required—this can help us identify the causes that lead to the failure of implants [7]. A study on magnesium biocompatible alloys showed that the addition of 2–3 wt.%Y in the Mg-0.5Ca alloy improved both the biodegradability rate and cytocompatibility behavior [8]. Duceac et al. demonstrated that ceftriaxone-loaded chitosan nanoparticles can be used as a carrier in antibiotic delivery [9].

Seifert et al. presented research, which furthered the understanding of some mechanisms: co-sputtered or multilayered Ti-Al films with a thickness of 200 nm were deposited on thermally oxidized Si substrates. It was concluded that in order to realize a high temperature stability of γ-TiAl thin films, a contact to SiO_2_ needs to be avoided by substituting the barrier with another material, e.g., AlN, or by using an additional protection layer. Also, a combined barrier layer consisting of 20 nm AlN and 20 nm SiO_2_ was able to prevent the oxidation of the RuAl film on CTGS up to 800 °C in air and 900 °C in HV. On LGS, stable films are realized up to 600 °C in air and 900 °C in HV [10,11].

Zinc oxide films were produced by means of the electron beam evaporation method; the ones produced at room temperature consisted of ZnO and Zn crystallites, and their optical constants exhibit a metallic-like behaviour [12].

Hashim et al. [13] demonstrated the effect of Ni on the suppression of Sn whisker formation in a Sn-0.7Cu solder joint, concluding that a small amount of Ni addition (~500 ppm) was able to alter the microstructure of Cu_6_Sn_5_ to form a (Cu,Ni)_6_Sn_5_ IMC intermetallic layer, and it is very significant to the nucleation and growth of Sn whiskers.

The performance of Sn-3.0Ag-0.5Cu composite solder with kaolin geopolymer ceramic reinforcement on microstructure and mechanical properties under isothermal ageing, resulting in the morphology of interfacial IMC layer of non-reinforced SAC305 and SAC305-KGC composite solder joints, showed a duplex IMC structure comprises of scallop-type Cu_6_Sn_5_ and layer-type Cu_3_Sn [14].

A study by Titu et al. [15] concluded that there is an economic advantage obtained when cutting semi-finished products of alloy steels by EDMCB, using the metal band as TO (tool).

A different approach was discussed in order to evaluate some medieval documents, a very important piece of our history [16]; the study revealed the morphological changes of parchment that occurred at various levels in the collagen fibrous mesh and established the state of conservation of the support, writing, and decorations, as well as the pigments involved.

All this published research will offer a new approach for further studies in order to create a sustainable society based on knowledge.

This issue is in memoriam Henriette Szilagyi, Cluj-Napoca Branch Director of the URBAN INCERC Institute, Romania.

## Data Availability

Not applicable.

## References

[1] Sandu A., Vasilache V., Sandu I., Sieliechi J., Kouame I., Matasaru P., Sandu I. (2019). Characterization of the Acid-Base Character of Burned Clay Ceramics Used for Water Decontamination. Materials.

[2] Nergis D.B., Abdullah M., Sandu A., Vizureanu P. (2020). XRD and TG-DTA Study of New Alkali Activated Materials Based on Fly Ash with Sand and Glass Powder. Materials.

[3] Azimi E., Abdullah M., Vizureanu P., Salleh M., Sandu A., Chaiprapa J., Yoriya S., Hussin K., Aziz I. (2020). Strength Development and Elemental Distribution of Dolomite/Fly Ash Geopolymer Composite under Elevated Temperature. Materials.

[4] Hegyi A., Lăzărescu A., Szilagyi H., Grebenişan E., Goia J., Mircea A. (2021). Influence of TiO_2_ Nanoparticles on the Resistance of Cementitious Composite Materials to the Action of Bacteria. Materials.

[5] Borlea (Mureşan) S., Tiuc A., Nemeş O., Vermeşan H., Vasile O. (2020). Innovative Use of Sheep Wool for Obtaining Materials with Improved Sound-Absorbing Properties. Materials.

[6] Rogale D., Majstorović G., Rogale S.F. (2020). Comparative Analysis of the Thermal Insulation of Multi-Layer Thermal Inserts in a Protective Jacket. Materials.

[7] Nica M., Cretu B., Ene D., Antoniac I., Gheorghita D., Ene R. (2020). Failure Analysis of Retrieved Osteosynthesis Implants. Materials.

[8] Istrate B., Munteanu C., Lupescu S., Chelariu R., Vlad M., Vizureanu P. (2020). Electrochemical Analysis and In Vitro Assay of Mg-0.5Ca-xY Biodegradable Alloys. Materials.

[9] Duceac L., Calin G., Eva L., Marcu C., Bogdan Goroftei E., Dabija M., Mitrea G., Luca A., Hanganu E., Gutu C. (2020). Third-Generation Cephalosporin-Loaded Chitosan Used to Limit Microorganisms Resistance. Materials.

[10] Seifert M. (2020). High Temperature Behavior of RuAl Thin Films on Piezoelectric CTGS and LGS Substrates. Materials.

[11] Seifert M., Lattner E., Menzel S., Oswald S., Gemming T. (2020). Phase Formation and High-Temperature Stability of Very Thin Co-Sputtered Ti-Al and Multilayered Ti/Al Films on Thermally Oxidized Si Substrates. Materials.

[12] Skowronski L., Ciesielski A., Olszewska A., Szczesny R., Naparty M., Trzcinski M., Bukaluk A. (2020). Microstructure and Optical Properties of E-Beam Evaporated Zinc Oxide Films—Effects of Decomposition and Surface Desorption. Materials.

[13] Hashim A., Salleh M., Sandu A., Ramli M., Yee K., Mohd Mokhtar N., Chaiprapa J. (2021). Effect of Ni on the Suppression of Sn Whisker Formation in Sn-0.7Cu Solder Joint. Materials.

[14] Zaimi N., Salleh M., Sandu A., Abdullah M., Saud N., Rahim S., Vizureanu P., Said R., Ramli M. (2021). Performance of Sn-3.0Ag-0.5Cu Composite Solder with Kaolin Geopolymer Ceramic Reinforcement on Microstructure and Mechanical Properties under Isothermal Ageing. Materials.

[15] Țîțu A., Vizureanu P., Țîțu Ș., Sandu A., Pop A., Bucur V., Ceocea C., Boroiu A. (2020). Experimental Research on the Cutting of Metal Materials by Electrical Discharge Machining with Contact Breaking with Metal Band as Transfer Object. Materials.

[16] Haulică M., Florescu O., Vasilache V., Sandu I. (2020). The Comparative Study of the State of Conservation of Two Medieval Documents on Parchment from Different Historical Periods. Materials.

